# *Metarhizium brunneum* Blastospore Pathogenesis in *Aedes aegypti* Larvae: Attack on Several Fronts Accelerates Mortality

**DOI:** 10.1371/journal.ppat.1005715

**Published:** 2016-07-07

**Authors:** Abeer M. Alkhaibari, Aline T. Carolino, Sare I. Yavasoglu, Thierry Maffeis, Thalles C. Mattoso, James C. Bull, Richard I. Samuels, Tariq M. Butt

**Affiliations:** 1 Department of Biosciences, College of Science, Swansea University, Singleton Park, Swansea, United Kingdom; 2 Department of Entomology and Plant Pathology, State University of North Fluminense, Campos dos Goytacazes, Rio de Janeiro, Brazil; 3 Department of Biology, Faculty of Arts & Sciences, Adnan Menderes University, Aydin, Turkey; 4 College of Engineering, Swansea University, Swansea, United Kingdom; Monash University, AUSTRALIA

## Abstract

*Aedes aegypti* is the vector of a wide range of diseases (e.g. yellow fever, dengue, Chikungunya and Zika) which impact on over half the world’s population. Entomopathogenic fungi such as *Metarhizium anisopliae* and *Beauveria bassiana* have been found to be highly efficacious in killing mosquito larvae but only now are the underlying mechanisms for pathogenesis being elucidated. Recently it was shown that conidia of *M*. *anisopliae* caused stress induced mortality in *Ae*. *aegypti* larvae, a different mode of pathogenicity to that normally seen in terrestrial hosts. Blastospores constitute a different form of inoculum produced by this fungus when cultured in liquid media and although blastospores are generally considered to be more virulent than conidia no evidence has been presented to explain why. In our study, using a range of biochemical, molecular and microscopy methods, the infection process of *Metarhizium brunneum* (formerly *M*. *anisopliae*) ARSEF 4556 blastospores was investigated. It appears that the blastospores, unlike conidia, readily adhere to and penetrate mosquito larval cuticle. The blastospores are readily ingested by the larvae but unlike the conidia are able infect the insect through the gut and rapidly invade the haemocoel. The fact that pathogenicity related genes were upregulated in blastospores exposed to larvae prior to invasion, suggests the fungus was detecting host derived cues. Similarly, immune and defence genes were upregulated in the host prior to infection suggesting mosquitoes were also able to detect pathogen-derived cues. The hydrophilic blastospores produce copious mucilage, which probably facilitates adhesion to the host but do not appear to depend on production of Pr1, a cuticle degrading subtilisin protease, for penetration since protease inhibitors did not significantly alter blastospore virulence. The fact the blastospores have multiple routes of entry (cuticle and gut) may explain why this form of the inoculum killed *Ae*. *aegypti* larvae in a relatively short time (12-24hrs), significantly quicker than when larvae were exposed to conidia. This study shows that selecting the appropriate form of inoculum is important for efficacious control of disease vectors such as *Ae*. *aegypti*.

## Author Summary

Mosquitoes transmit a range of diseases which have a profound impact on human health. *Aedes aegypti* vectors dengue, one of the fastest emerging diseases and, more recently, the Zika virus, which has been linked to thousands of birth defects over the last two years in Brazil. Insect pathogenic fungi such as *Metarhizium brunneum* are effective in killing mosquito adults and larvae. They exhibit much plasticity, producing aerial conidia on solid substrates and blastospores in liquid media. We not only show that blastospores are more virulent than conidia but present evidence explaining why they are more aggressive. The blastospore mode of pathogenesis differs from that of conidia in several ways. Firstly, blastospores appear to be more dependent on entry using mechanical force than by secretion of cuticle degrading proteases such as Pr1. Blastospores produce copious mucilage which ensures that many spores attach to the cuticle. They are also readily ingested and able to penetrate the gut wall rapidly and colonize the haemocoel. Multiple entry points and gross damage to the cuticle and gut results in rapid larval death. Conidia neither adhere to the cuticle nor germinate in the gut but cause Pr1 stress induced mortality, which takes a slightly longer time. Blastopores, therefore, have greater potential for the control of *Ae*. *aegypti* larvae in mosquito control programmes

## Introduction

*Aedes aegypti* is the vector of a wide range of viral diseases (e.g. yellow fever, dengue, Chikungunya and Zika) [1–5]. Dengue fever annually affects 284 to 528 million people around the world [6]. The range of this pest appears to be expanding due to global warming [7]. Of major concern is the establishment of *Ae*. *aegypti* and *Aedes albopictus* throughout Europe with the latter now firmly established in Southern Europe [7]. The success of these two species is partly due to their ability to readily adapt to urban environments and the tolerance of the eggs to desiccation [8]. Current control is still heavily dependent upon the use of chemical pesticides, which should be discouraged because of the risks they pose to human health and the environment [9, 10]. Moreover, mosquitoes are also rapidly developing resistance to chemical insecticides as well as to the biological larvicide *Bacillus thuringiensis* [11–14]. Much attention is currently being focussed on the use of entomopathogenic fungi (EPF) such as *Beauveria bassiana* and *Metarhizium anisopliae* for the control of mosquito adults and larvae [15–24] as they are considered to be environmentally friendly and highly versatile [25].

Both aerial conidia and blastospores are highly efficacious in killing mosquito larvae [26–28]. Blastospores differ from conidia in several ways. The former are thin-walled, pleomorphic, hydrophilic spores produced relatively inexpensively due to short fermentation times within 2–3 days in liquid media, whereas conidia are uniform shaped, hydrophobic spores produced within 12–20 days on solid substrates such as rice [28, 29]. Although aerial conidia have a comparatively longer shelf life, blastospores are normally considered more virulent against susceptible hosts [28–38]. Exactly why blastospores are more aggressive is unclear. Blastospores generally germinate faster than conidia (2-8hrs versus 12–24 hrs) and this attribute is considered to be a virulence determinant [29, 39]. Slower germination means longer exposure of propagules to deleterious biotic (e.g. antagonistic microbes) and abiotic (e.g. humidity, UV, temperature) factors that negatively affect propagule viability [40, 41]. Furthermore, it gives the host more time to mobilise its defences and resist infection [42, 43]. In the aquatic environment, blastospores of *B*. *bassiana*, *Tolypocladium cylindrosporum* and *M*. *anisopliae* were found to be more virulent against mosquito larvae than aerial conidia [27, 28, 44]. According to Miranpuri and Khachatourians [28] the primary infection sites of *B*. *bassiana* blastospores were the head and the anal region, although the most preferred site for invasion was the larval gut. However, none of these studies provided an explanation as to why the blastospores were more virulent than conidia. Both conidia and blastospores adhere to the surface of terrestrial arthropod hosts and penetrate the cuticle using a combination of enzymes and mechanical force [45, 46]. Recent studies have shown that the mode of *M*. *anisopliae* pathogenesis against *Ae*. *aegypti* larvae was radically different from that observed when attacking terrestrial hosts in that the conidia failed to adhere to the cuticle surface and that death was due to stress induced in the insect gut by the spore bound proteases on the surface of ingested conidia [47, 48]. Furthermore, the ingested conidia did not germinate and colonise the haemocoel but remained confined to the gut lumen [47]. It was postulated that *M*. *anisopliae*, which is normally found in the soil, has not evolved to infect aquatic invertebrates, hence the atypical mode of pathogenesis. To date, there has been no detailed study of the infection processes of EPF blastospores. Some fluorescence and ultrastructural studies of EPF blastospores have been conducted but mostly of those infecting terrestrial insects [49, 50]. The current multidisciplinary study investigates various aspects of *Metarhizium brunneum* blastospore pathogenesis in *Ae*. *aegypti* larvae with the goal of understanding the infection process when compared to that observed in terrestrial hosts. The current study establishes why blastospores were more virulent than conidia and are highly interesting candidates for vector control, given that mortality was observed in hours rather than days. The significance of these findings as regards the use of *Metarhizium* for mosquito control is discussed.

## Results

### Comparison of blastospore and conidial virulence

**Fig 1:**
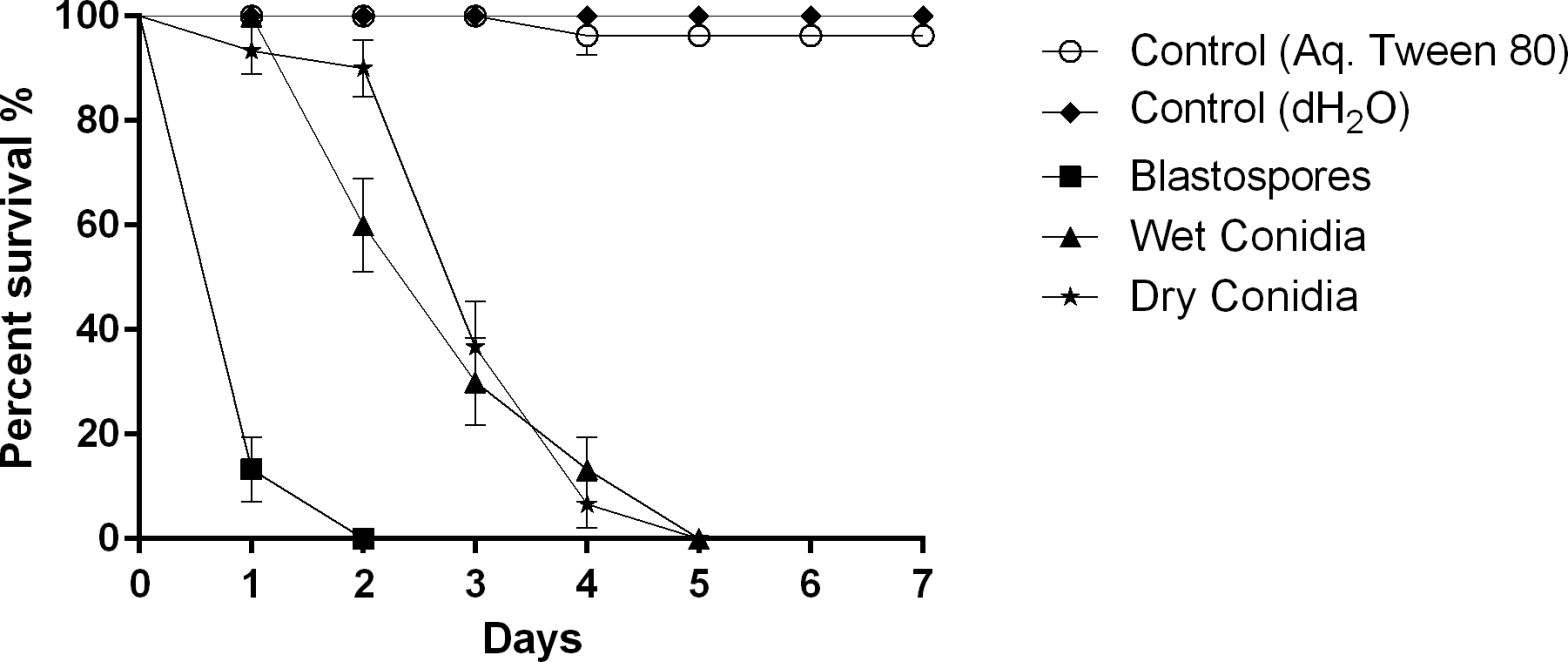
Susceptibility of *Aedes aegypti* larvae to blastospores, wet and dry conidia of *Metarhizium brunneum* ARSEF 4556. Survival curves of *Ae*. *aegypti* (3^rd^-4^th^) instar larvae (n = 30 per treatment) inoculated with blastospores or conidia (wet and dry) of *M*. *brunneum* ARSEF 4556 applied at final dose of 10^7^ spores ml^-1^. Blastospores significantly decreased survival compared to all other treatments (p < 0.001), but there were no statistical differences in the survival when comparing wet and dry conidia. The negative controls were either distilled water or 0.05% aqueous Tween. Error is represented as SE.

Significant differences in survival were observed among treatments (χ^2^ = 163.7, df = 3, overall *P*<0.001, Fig 1). Blastospores of *M*. *brunneum* ARSEF 4556 were significantly more virulent against *Ae*. *aegypti* larvae than either the wet (χ^2^ = 49.13, pairwise *P*<0.001) or dry (χ^2^ = 55.32, *P*<0.001) conidial formulations (Fig 1). There was no significant difference in survival between the wet and dry conidia (χ^2^ = 0.568, *P* = 0.451, Fig 1). Blastospores caused 100% mortality 2 days post inoculation (pi) (LT_50_ = 0.92 days), while wet and dry conidia only caused 100% mortality at 5 days pi with LT_50_ values of 2.52 and 2.76 days respectively (Fig 1).

### Protease activity during blastospore pathogenesis

**Fig 2:**
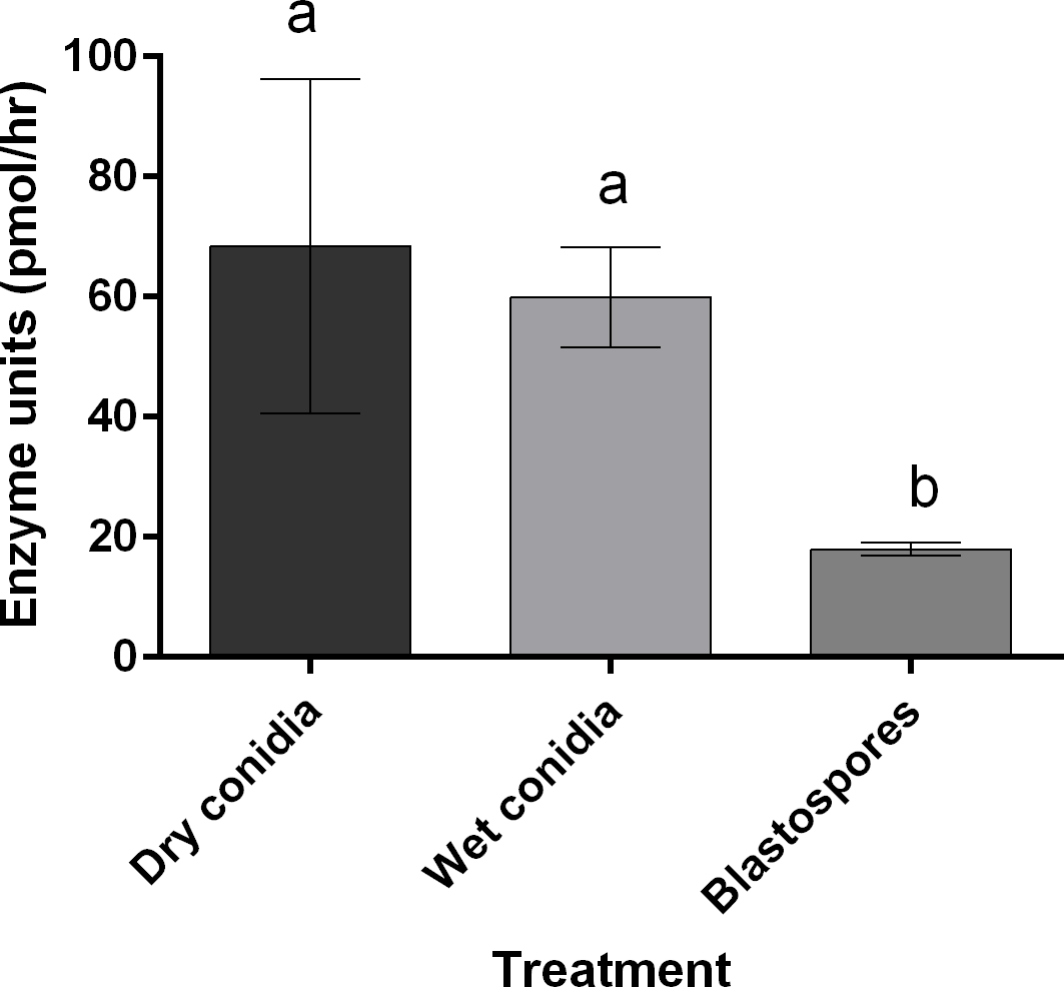
Comparison of *Pr1* enzyme activity between blastospores, wet and dry conidia of *Metarhizium brunneum* ARSEF 4556. Activity of *Pr1* bound to the cell wall of conidia (dry and wet) and blastospores in culture media. Letters denote statistical differences. Error is represented as 95% ci.

**Fig 3:**
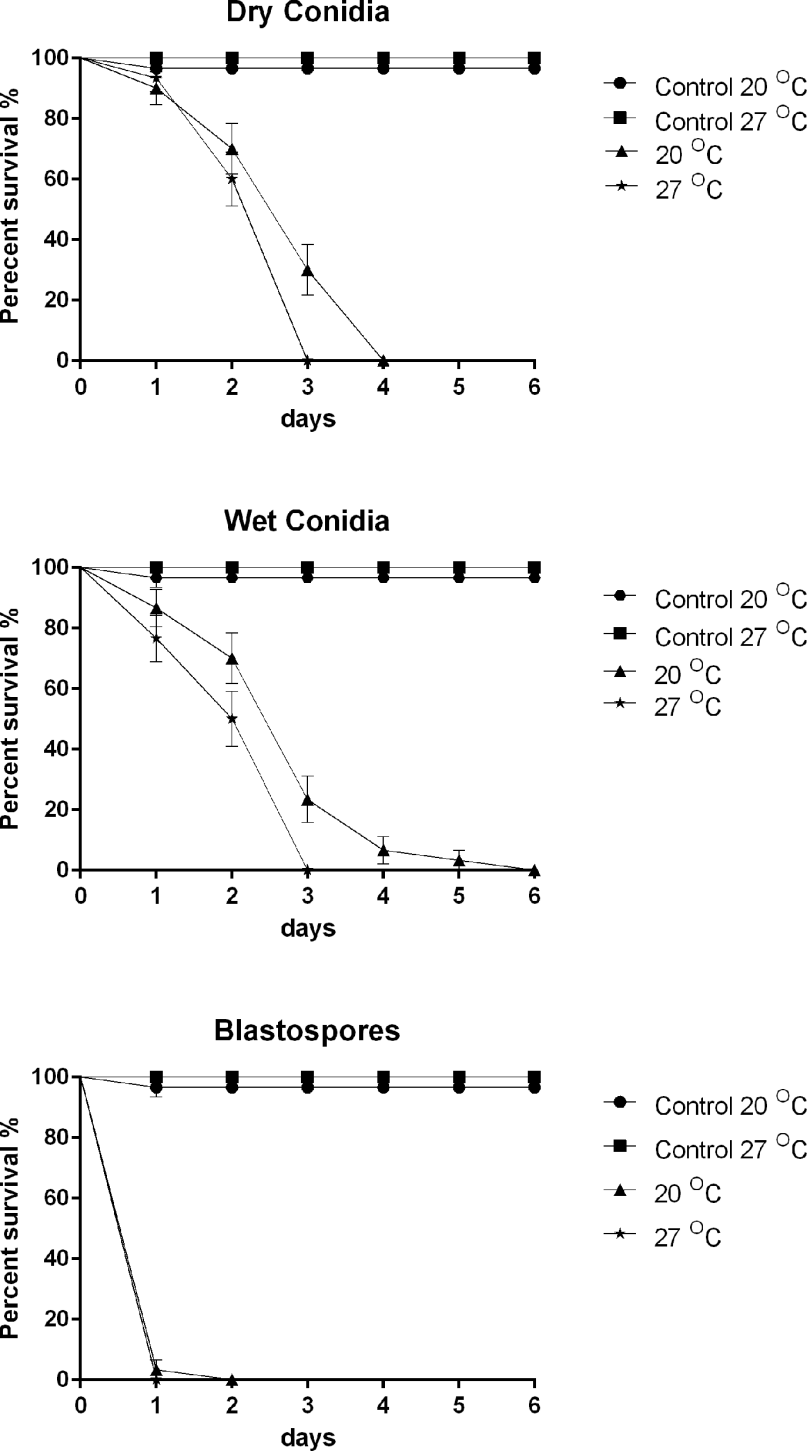
Survival of *Aedes aegypti* larvae exposed to conidia or blastospores at 20°C and 27°C. Survival curves of *Ae*. *aegypti* larvae (n = 30) exposed to conidia (wet & dry) and blastospores of *M*. *brunneum* at a concentration of 10^7^ spores ml^-1^ incubated at 20°C and 27°C. Survival was similar for larvae inoculated with blastospores at 20°C or 27°C (*P* = 0.317) but significant differences were observed for larvae exposed to either dry (*P* = 0.022) or wet (*P* = 0.011) conidia when comparing these two temperatures. Controls consisted of distilled water or 0.05% aqueous Tween. Error is represented as SE.

**Fig 4:**
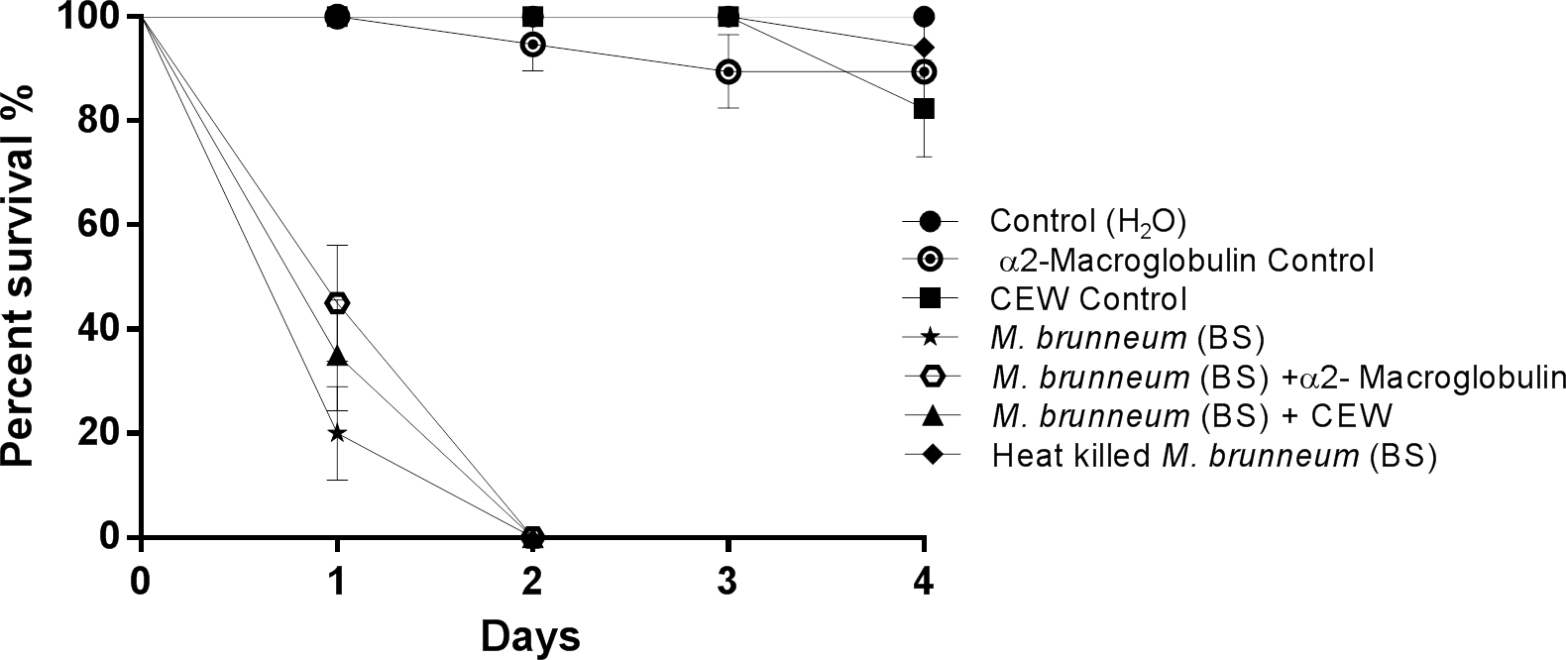
Survival of *Aedes aegypti* larvae exposed to blastospores in the presence of different protease inhibitors. *Ae*. *aegypti* larvae (n = 72) were exposed to *M*. *brunneum* blastospores with and without the addition of the protease inhibitors chicken egg white (CEW, a Pr1 specific inhibitor) and α2-macroglobulin (global protease inhibitor). The Kaplan-Meier method was used to plot survival curves of larvae; Log-rank test was used to assess difference in survival between treatments. Controls consisted of either distilled water or distilled water with protease inhibitors. Error is represented as SE.

Three lines of evidence are presented which show that proteases are not critical virulence determinants during blastospore infection. Firstly, the level of Pr1 associated with blastospore pellets was significantly lower than for wet (diff = 41.97 [95% c.i.: lower = 17.55, upper = 66.38], *P* = 0.003) or dry conidia (50.49 [26.08, 74.90], *P* = 0.001) as shown in Fig 2. Secondly, conducting assays at increased temperatures did not accelerate larval mortality with blastospores (χ^2^ = 1.000, df = 3, *P* = 0.317), where the respective morality rates from blastospore infection were 97% and 100% at 20°C or 27°C 24 hrs pi (Fig 3). These values are similar to those observed at 25°C (Fig 1). In contrast, conidia (wet/dry) bound proteolytic activity increased with temperature and corresponding mortality rates of larvae inoculated with dry or wet conidia were significantly higher at 27°C than 20°C as shown in Fig 3 (Dry: χ^2^ = 5.214, df = 3, *P* = 0.022; Wet: χ^2^ = 6.513, df = 3, *P* = 0.011). Finally, protease inhibitors did not influence blastospore virulence (Fig 4) whereas they greatly affected conidial virulence [47]. Although mortality appeared to be slightly lower in suspensions containing protease inhibitors 24 hr pi, there were no statistically significant differences in virulence between *M*. *brunneum* untreated blastospores when compared to blastospores treated with α2-macroglobulin (χ^2^ 2.778, df = 6, *P* = 0.096) or chicken egg white inhibitor (χ^2^ = 1.100, df = 6, *P* = 0.294) as seen in Fig 4. No differences in larval survival were observed when using α2-macroglobulin and chicken egg white inhibitors (Fig 4: χ^2^ = 0.406, df = 6, *P* = 0.524,). Furthermore, there were no significant differences in survival between larvae exposed to heat killed blastospores and the untreated control (Fig 4: χ^2^ = 1.000, df = 6, *P* = 0.317).

### Interaction of blastospores with the integument and intestine

**Fig 5:**
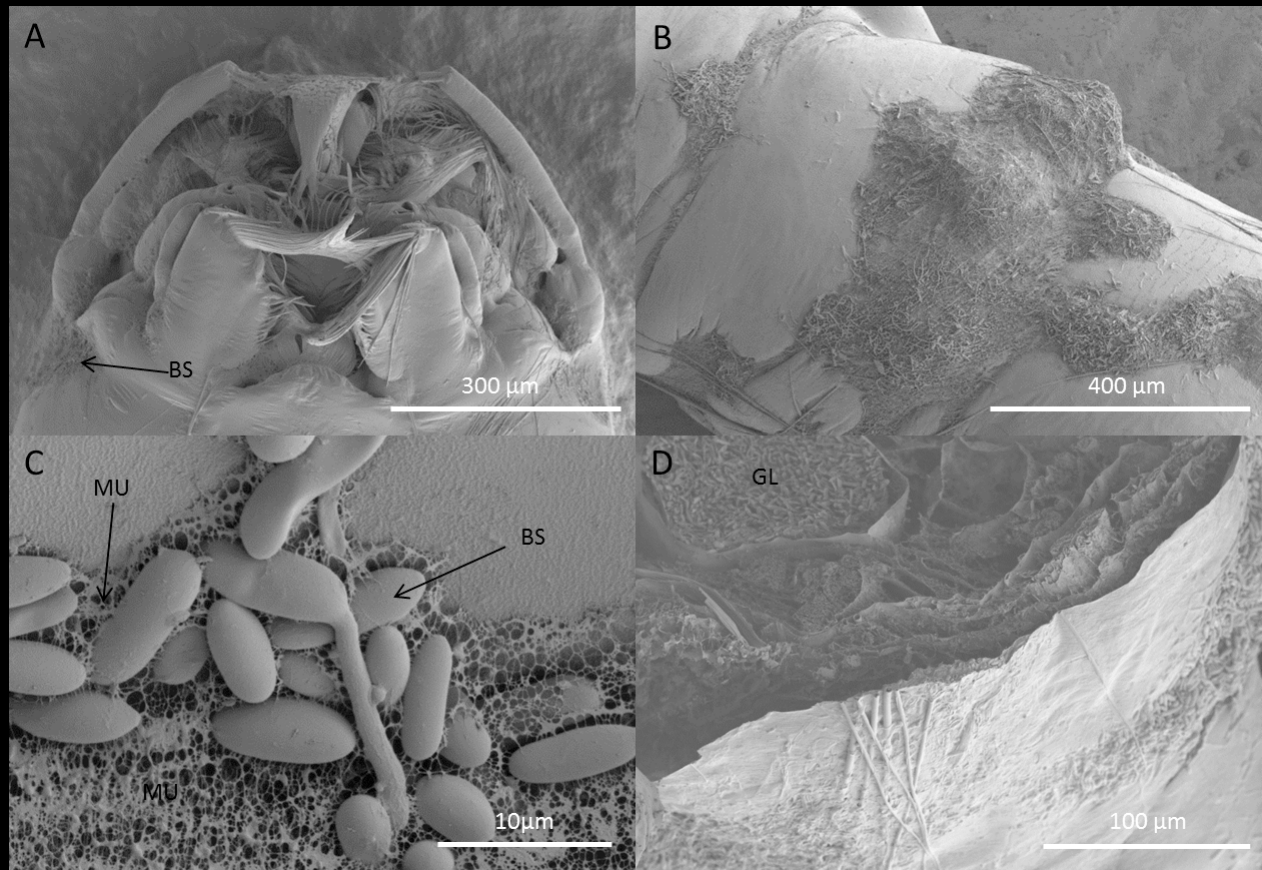
Scanning electron microscopy of *Aedes aegypti* larvae infected with *Metarhizium brunneum* blastospores. Larvae were inoculated with 1X10^7^ blastospores ml^-1^ and prepared for SEM 20 hrs post inoculation. (A): Head of *Ae*. *aegypti* larva showing blastospores (BS) attached to the surface of the cuticle. (B): Blastospores at different stages of germination attached to surface of abdomen. (C): Germinating and non-germinating blastospores surrounded by a mucilaginous matrix (M). (D). Cross section of infected larva showing blastospores of *M*. *brunneum* occluding the gut lumen (GL).

**Fig 6:**
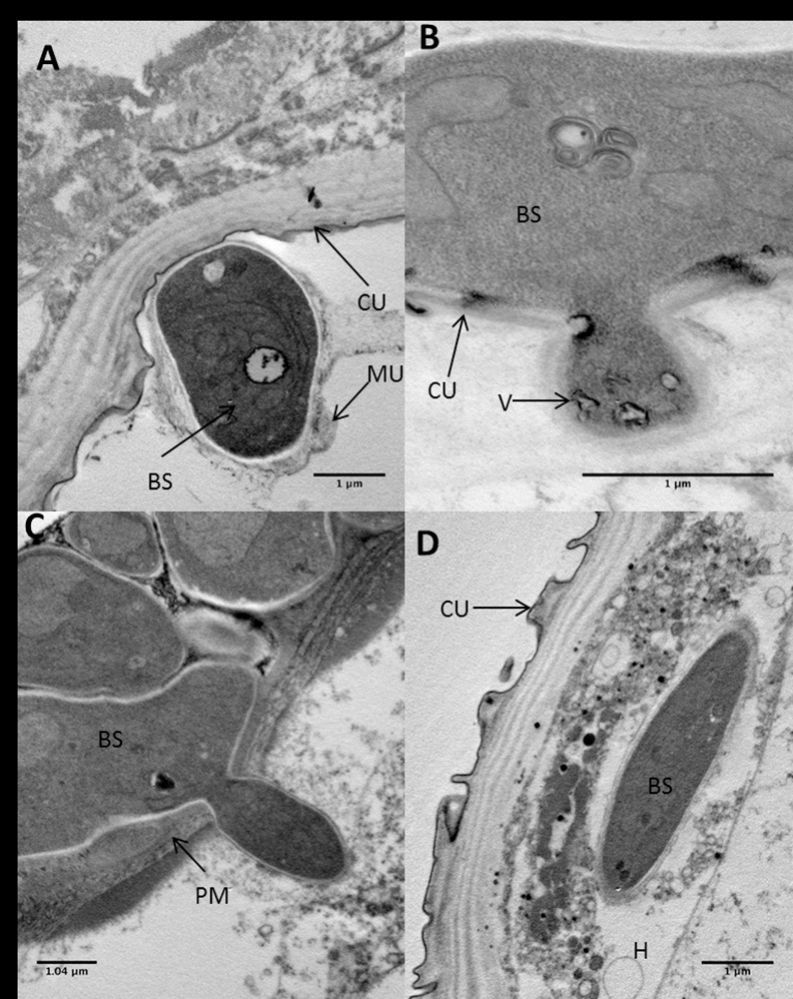
Transmission electron microscopy of *Aedes aegypti* larvae infected with *Metarhizium brunneum* blastospores 24 hr post-inoculation. (A) Blastospore (BS) firmly adhering to surface of the cuticle (CU). The blastospore has dense cytoplasm, a relatively thin wall and a coating of mucilage (MU) which extends beyond the fungus. The mucilage consists of heterogeneous electron opaque material. (B) Penetration of the larval cuticle. Vacuoles (V) containing electron dense material evident in the penetration hyphae. Cuticle readily distorted by penetration hypha. (C) Blastospores in gut lumen penetrating the peritrophic membrane (PM). (D) One blastospore has penetrated the midgut epithelium and has entered the haemocoel (H).

Blastospores of *M*. *brunneum* adhered to almost any part of the mosquito larval cuticle (Fig 5A and 5B). There appeared to be no blockage of the mouthparts or siphons (Fig 5A). Blastospores often formed clumps but they were also present as individual propagules (Fig 5C). Low temperature-scanning electron microscopy (SEM) showed that the blastospores were often covered with copious mucilage which was present in sheet, reticulate and strand form (Fig 5C). The mucilage appeared to be water insoluble since it was present when larvae were recovered from water. Mucilage strands were extruded at the fungus-cuticle interface and were particularly abundant at blastospore apices (Fig 5C). Mucilage strands were strong as they resisted destruction when samples were plunged in the preparatory pre-cooled nitrogen slush and their structure was not affected by the solvents used during sample preparation for transmission electron microscopy (TEM). In thin sections, mucilage appeared as an amorphous, non-uniform, matrix of fibrils that coated the relatively thin cell wall but also extended beyond the blastospores (Fig 6A).

Most blastospores appeared to be turgid, cylindrical cells with a smooth surface (Fig 5C). They grew over the surface of the cuticle but not extensively with little evidence of branching. Blastospores rarely produced appressoria, with penetration pegs being produced sub-apically (Fig 6B). The relatively short, narrow penetration pegs quickly expanded within the cuticle or soon after breaching the cuticle (Fig 6B). The peg retained a relatively thin cell wall which was covered with an amorphous matrix (Fig 6A). There was no obvious clearing around the peg or gross distortion of the cuticle at the penetration site.

**Fig 7:**
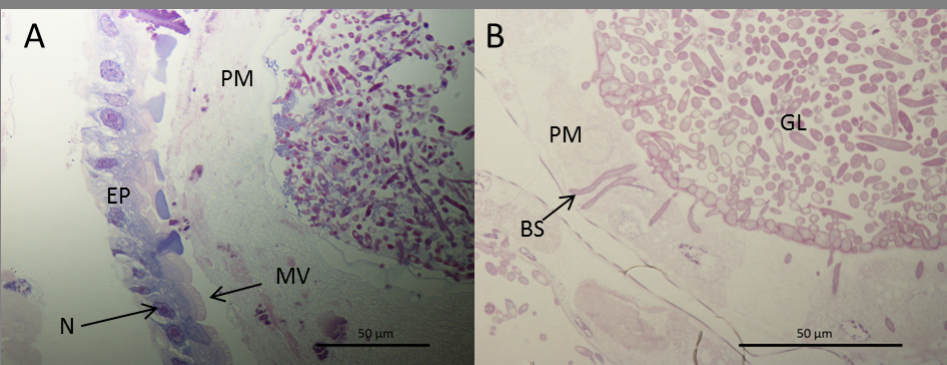
Cross section of *Aedes aegypti* larvae 24 hrs post infection with *Metarhizium brunneum* blastospores. (A) Blastospores of *M*. *brunneum* mostly confined to gut lumen. (B) Blastospores adjacent to the peritrophic membrane are swollen with some having penetrated the peritrophic membrane and midgut epithelium. Cells colonizing the haemocoel consisted of short filaments or hyphal bodies as well as yeast like cells. There was no evidence of branched filamentous hyphae. EP: Epithelium, PM: peritrophic membrane, MV: Microvilli, N: Nuclei, BS: Blastospores, GL: Gut lumen.

Both SEM and light microscopy confirmed that blastospores were ingested by the larvae. Numerous blastospores were present in the gut lumen (Figs 5D and 7). Many cells adhered to the peritrophic membrane, these became swollen and ultimately gave rise to penetration hyphae which penetrated the peritrophic membrane and eventually the midgut epithelium (Fig 6C). The blastospores colonizing the haemocoel after breaching the gut were similar in phenotype to those produced in liquid media (Fig 7).

### Blastospore structure

TEM revealed that each blastospore contained dense cytoplasm, several nuclei and mitochondria but very few vacuoles. Most of the vacuoles were relatively small, containing electron opaque material. This material appeared to be extruded through the cell wall and deposited between the fungus and host cuticle (Fig 6B). The electron opaque material was associated with individual blastospores as well as blastospores in groups at the cuticle surface. Electron opaque material was also observed associated with ingested blastospores in the gut lumen (Fig 6C). The distribution of this material was not extensive or uniform.

The cytoplasm retained a dense appearance irrespective of whether the blastospores grew on the host surface, penetrating the cuticle or midgut epithelium or when colonizing the haemocoel. Blastospores appeared to divide by budding but the size of the daughter cells varied before breakage from the mother cell. In some instances the daughter cells appeared as short filaments (Fig 7).

**Fig 8:**
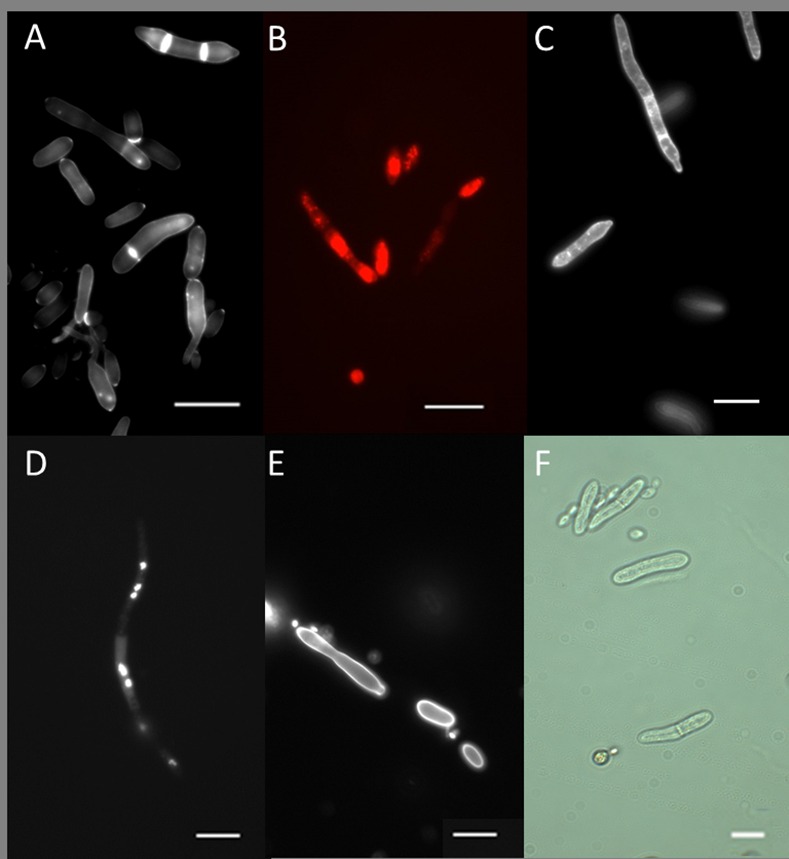
Fluorescence microscopy of *Metarhizium brunneum* blastospores. **(A)** Calcofluor White staining of blastospores. Cell walls fluoresced weakly except at apices and septa. (B) Rhodamine 123 was used to visualise mitochondria in blastospores. (C) Filipin staining of ergosterol present in the plasma membrane. (D) Fluorescent staining of nuclei with DAPI. (E) FITC staining of proteins (F) Blastospores as seen in bright-field (F). Scale bar = 5 μm.

A striking feature of blastospores was the poor staining of the cell wall with calcofluor (Fig 8). Both incipient and fully formed septa fluoresced intensely. Intense fluorescent spots were observed at the apices of some blastospores and occasionally along the length of the cell (Fig 8). The pattern of staining was similar whether the blastospores were produced *in vitro* or in *vivo*. The plasma membrane stained weakly with filipin, fluorescence being intense at septa and cell apices (Fig 8). Fluorescent spots were also observed in a number of cells (Fig 8).

### Expression of *Metarhizium* pathogenicity related genes

**Fig 9:**
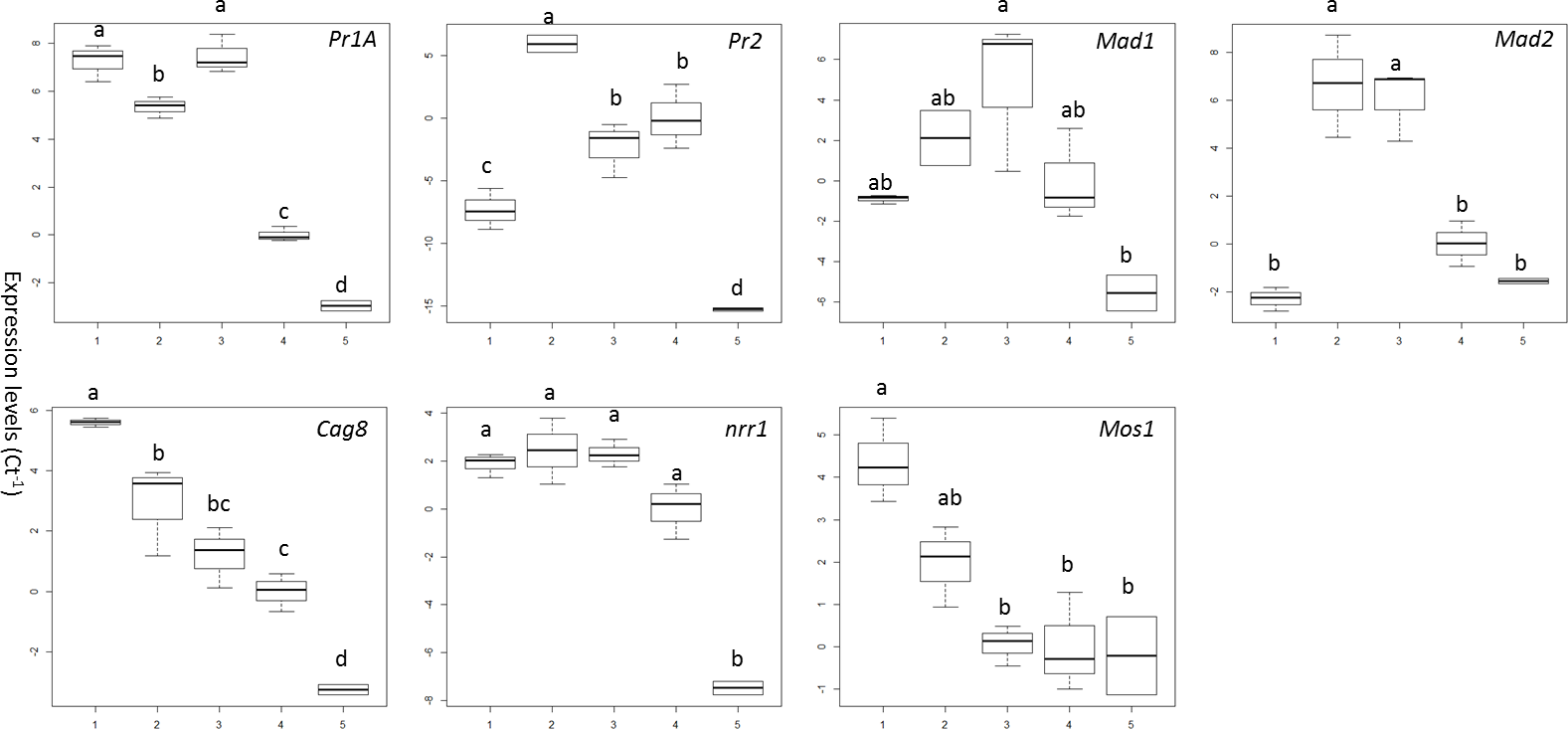
Expression of pathogenicity related genes in *Metarhizium brunneum* blastospores 20.3 hours post-infection. Gene expression analysed by quantitative PCR included: proteases (*Pr1A*, *Pr2*), adhesins (*Mad1*, *Mad2*), an osmosensor (*Mos1*),regulators of G-protein signalling (*Cag8) and* nitrogen (*nrr1)*. X axis shows: 1: infected living larvae, 2: infected dead larvae, 3: blastospores in the presence of *Ae*. *aegypti* larvae 4: blastospores in absence of the larvae, 5: *Tenebrio molitor* (terrestrial host) positive control. Boxes denote interquartile range, bisected horizontally by median values; whiskers extend to 1.5× interquartile range beyond boxes; outliers are marked as dots beyond whiskers. Expression is shown as the inverse of the number of amplification cycles to reach Critical Threshold values (CT^-1^).

Expression of genes directly and indirectly linked with pathogenesis namely *Pr1*, *Pr2*, *Mad1*, *Mad2*, *Mos1*, *Cag8*, and *nrr1* was generally higher in blastospores in the aquatic setting (i.e. in infected larvae or in the presence or absence of the larvae) than in *Tenebrio*, the terrestrial positive control host (Fig 9). Expression of the cuticle degrading enzyme *Pr1* was highest in blastospores in the presence of larvae (i.e. blastospores that were still in suspension), which was statistically similar to blastospores in infected but live larvae (diff = -0.198 [95% c.i.: lower = -1.837, upper = 1.440], Tukey’s HSD: *P* = 0.993). Expression was slightly lower in infected dead insects but much lower in blastospores suspended in water in the absence of *Aedes* larvae (Fig 9). Expression of the protease *Pr2* was highest in dead infected larvae but with similar levels of expression when comparing blastospores in the presence and absence of larvae (-2.320 [-7.699, 3.060], *P* = 0.595). In infected live larvae, *Pr2* expression was the lowest among all treatments excluding *Tenebrio* (Fig 9). Most notably there was an elevated expression of the adhesion genes *Mad1* and *Mad2*. *Mad 1* expression was highest in *M*. *brunneum* 4556 blastospores in the presence of larvae (Fig 9). However, this was not significantly different to infected live larvae (-5.735 [-12.39, 0.924], *P* = 0.096), dead infected larvae (-2.706 [-10.15, 4.739], *P* = 0.723), and blastospores in absence of larvae (4.833 [-1.826, 11.49], *P* = 0.181, Fig 9). Expression of *Mad2* was equally high in infected dead larvae and blastospores in presence of live larvae (0.590 [-3.066, 4.245], *P* = 0.980). Similar expression levels of *Mad2* were observed in infected live larvae, blastospores in the absence of larvae and *Tenebrio* (Fig 9).

Expression of *Cag8* and *Mos1* was highest in live infected larvae followed by dead infected larvae. *Cag8* was higher in blastospores in the presence of live larvae than in their absence. *Mos1* was lowest in blastospores whether in the presence or absence of live larvae but similar to blastospores infecting *Tenebrio* (Fig 9). Expression of the nitrogen regulator gene *nrr1* was high in blastospores in all treatments except for *Tenebrio* (Fig 9).

### Expression of *Ae*. *aegypti* immune and stress management genes during infection

**Fig 10:**
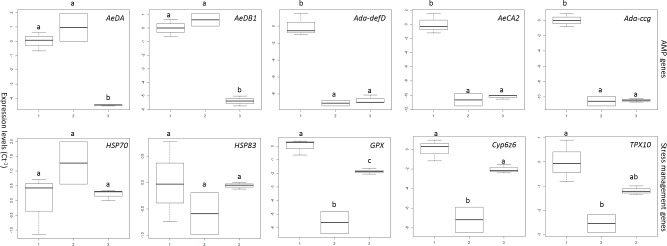
Expression of *Aedes aegypti* antimicrobial peptides (AMP) and stress management genes during infection with *Metarhizium brunneum* blastospores. Quantitative real time PCR used to analyse expression of stress management and AMP genes in *Ae*. *aegypti* larvae inoculated with *M*. *brunneum* blastospores at **1** *=* 0hr, **2** = 12hr and **3** = 20:30 hrs post-inoculation. AMP genes included; *AeDA* (Defensin A), *AeDB1* (Defensin B), *Ada-defD* (Defensin D), *AeCA2* (Cecropin A), and *Ada-ccg* (Cecropin G). Stress management genes include; *HSP70* (Heatshock protein 70), *HSP83 (*Heatshock protein 83), *GPX* (Glutathione peroxidase), *Cyp6Z6* (Cytochrome P450), and *TPX10* (Thiol peroxidase 10). Boxes denote interquartile range, bisected horizontally by median values; whiskers extend to 1.5× interquartile range beyond boxes; outliers are marked as dots beyond whiskers. Expression is shown as the inverse of number of amplification cycles to reach Critical Threshold values (CT^-1^).

The immune defence response of *Ae*. *aegypti* larvae was rapid following exposure to *M*. *brunneum* AFSEF 4556 blastospores; expression of all five AMPs (*AeDA*, *AeDB1*, *Ada-defD*, *AeCA2*, *Ada-ccg*) was elevated immediately after exposure to the pathogen (Fig 10). However, at 12 hrs pi only the expression of *AeDA*, *AeDB1* remained high. However by 20.5hrs pi, all the AMP genes were down regulated (Fig 10). The expression level of *AeDA*, *AeDB1* at 20.5 pi was significantly different in comparison with the control (*AeDA*: -4.468 [-6.428, -2.508], *P* = 0.002), (*AeDB1*: -5.375 [-6.803, -3.947], *P* < 0.001), or 12 hrs pi (*AeDA*: -5.428 [-7.619, -3.236], *P* = 0.001), (*AeDB1*: -5.985 [-7.581, -4.388], *P* < 0.001), where the gene expression was down regulated, with no difference between the control and 12hrs pi (*AeDA*: 0.960 [-1.232, 3.151], *P* = 0.398), (*AeDB1*: 0.610 [-0.987, 2.206], *P* = 0.481). Expression of *Ada-defD*, *AeCA2* and *Ada-ccg* was significantly lower than the controls at 12 hrs (*Ada-defD*: -9.078 [-11.79, -6.371], *P* < 0.001), (*AeCA2*: -10.67 [-13.79, -7.548], *P* < 0.001), (*Ada-ccg*: -10.54 [-12.59, -8.496], *P* < 0.001) and 20.5 hrs pi (*Ada-defD*: -8.708 [-11.13, -6.286], *P* < 0.001), (*AeCA2*: *-*10.215 [-13.01, -7.424], *P* < 0.001), (*Ada-ccg*: -10.43 [-12.26, -8.600], *P* < 0.001). However, between 12hrs and 20.5 hrs pi there was no significant difference (*Ada-defD*: 0.370 [-2.337, 3.078], *P* = 0.899), (*AeCA2*: 0.454 [-2.667, 3.574], *P* = 0.887), (*Ada-ccg*: 0.112 [-1.935, 2.159], *P* = 0.983).

Expression of five genes associated with stress management (*HSP70*, *HSP83*, *GPX*, *Cyp6Z6*, *TPX10)* showed an unusual disjointed pattern of expression (Fig 10). Expression of the heat shock genes *HSP70* and *HSP83* was similar at all three time points (*HSP70*: F_2,5_ = 1.701, *P* = 0.273), (*HSP83*: F_2,5_ = 0.811, *P* = 0.495). Expression of *GPX*, *Cyp6Z6*, and *TPX10* was high at 0hrs pi but was low at 12 hrs pi. All three genes appeared to be up-regulated at 20.5hrs pi. The level of *GPX* at 20.5 hr pi was significantly higher than at 12 hrs pi (diff = 3.754 [95% c.i.: lower = 1.844, upper = 5.663], Tukey’s HSD: *P* = 0.003), but it was significantly lower than in non-infected larvae (-1.1858 [-3.566,-0.150], *P* = 0.037). Expression of *Cyp6Z6* and *TPX 10* was significantly lower at 12 hrs pi than in the controls (*Cyp6Z6*: -7.178 [-10.46, -3.893], *P* = 0.002), (*TPX 10*: -2.543 [-4.330, -0.757], *P* = 0.013).

## Discussion

Blastospores of *M*. *brunneum* strain ARSEF 4556 were significantly more virulent than conidia in killing *Ae*. *aegypti* larvae. This study shows for the first time that blastospores have specific characteristics which explain why they are more aggressive to mosquito larvae than conidia. These attributes include the ability to readily adhere to the cuticle surface, production of copious mucilage and the ability to infect without the differentiation of appressoria or excessive production of the proteases Pr1 and Pr2. Blastospores infected larvae through integument and gut, retaining the dynamic blastospore form both on the host surface and during invasion of the haemocoel. This study also shows that the host immune and stress management responses rapidly kick in but are inadequate as they fail to prevent infection.

One of the most striking features of *M*. *brunneum* blastospores is their ability to adhere to the surface of the mosquito larval cuticle whereas conidia of the same fungus are unable to do so [47, 48]. Blastospores are hydrophilic, they readily suspend in water and have high affinity for hydrophilic surfaces [51]. This study shows for the first time that *M*. *brunneum* blastospores produce copious, robust, water insoluble mucilage, which may not only explain why these cells tended to clump but also firmly adhere to the mosquito larval surface in an aquatic environment. Exactly why the blastospores expend resources and energy in mucilage production is unclear. Fungi produce an extracellular matrix for a variety of reasons including adhesion of conidia, hyphae, appressoria and blastospores to host surfaces, protection against harmful UV radiation and creation of environments conducive for cuticle degrading enzyme activity [52]. There appears to be a relationship between mucilage production and hydrophilicity since several EPF species that produce wettable conidia often possess a mucilaginous coat [53].

Expression of the adhesin *Mad1* gene was elevated in blastospores exposed to *Ae*. *aegypti* larvae. This corroborates the findings of Barelli *et al*. [54] who found that *Mad1* was upregulated in the presence of insect cuticle. Disruption of *Mad1* in *M*. *anisopliae* delays germination, suppresses blastospore formation, and greatly reduces virulence [55]. Sustained expression of *Mad1* may explain why *M*. *brunneum* retained the blastospore form during invasion and colonisation of *Ae*. *aegypti* larvae. In contrast, *Mad2* expression was high in dead infected *Ae*. *aegypti* larvae and blastospores in the presence of larvae. *Mad2* is associated with adhesion to plant surfaces; disruption of this gene, which is under stress control, blocks adhesion of *M*. *anisopliae* to plant surfaces but has no effect on fungal differentiation or virulence [55]. Expression of *Mad2* as seen here, suggests that the gene was under starvation stress control as previously reported by Barelli and colleagues [54].

It was not possible to determine the chemical composition of the mucilage but it was clear from the SEM and TEM studies that the mucilage was highly resilient and that it contained feint electron opaque fibrils. Similar material has been reported associated with appressoria of several plant pathogenic fungi [56]. None of the fluorochromes tested stained the mucilage. Calcofluor only weakly stained the cell wall of *M*. *brunneum* blastospores suggesting that its composition was substantially different to that of the septa, bud scars and growing points which fluoresced intensely. Presumably the blastospore cell wall lacked β-glucans or that these sugars were masked by mucilage. Several researchers have reported that blastospores growing *in vivo* possess fewer carbohydrate epitopes than conidia or blastopores incubated in artificial culture media [57, 58]. β-glucans are powerful elicitors of the insect immune system [59]. Absence or masking of β-glucans may be an adaptation to avoid recognition by the host’s immune defences and may explain why no significant cellular or humoral defence responses were observed in *Aedes* larvae. Expression of AMPs was elevated immediately after exposure to the inoculum but not later suggesting that water soluble factors were being detected by the host, eliciting an immune response.

Another novel finding of this study was the failure of protease inhibitors to prevent blastospores from causing larval mortality even though the *Pr1* and *Pr2* cuticle degrading protease genes were expressed in blastospores. Pr1 is a virulence determinant of terrestrial arthropod hosts [60] and can cause stress induced mortality in *Ae*. *aegypti* larvae following ingestion of viable conidia [47]. It is tempting to speculate that blastospores are therefore more dependent upon mechanical forces or enzymes other than Pr1 to penetrate the larval cuticle and peritrophic membrane. The current observations and the fact that EPF are able to penetrate non-host plant cells and inert substrates, suggest that mechanical force may be more important than previously realised [61, 62].

The highest level of expression of *Cag8*, *nrr1*, and *Mos1* was detected in infected living larvae, presumably their concerted activity enables *M*. *brunneum* blastospores to adapt to the host and cope with disparate stresses encountered particularly at the cuticle surface and gut lumen [63]. *Mos1* plays a role in fungal differentiation (appressoria, hyphal bodies) and ability to cope with stress (osmotic, oxidative). Inactivation of this gene reduces virulence [55]. Although *M*. *brunneum* blastospores failed to readily produce appressoria, *Mos1* probably plays a role in the infection process such as maintaining cell turgor and stress management since the level of expression declines in dead infected insects. *Cag8* plays a role in the regulation of conidiation, virulence and hydrophobin synthesis [64]. Disruption of this gene can result in the production of irregular shaped blastospores and a decline in virulence [63]. In the current study this gene may be involved in the retention of the blastospores phenotype and contribute to its virulence, hence the elevated activity during infection in the live insects but a decline in dead infected insects.

*Pr1* and *Pr2* are subject to nitrogen derepression by *nrr1*. The *nrr1* protein binds to GATA sites from the promoter region of the *Pr1* gene in *M*. *anisopliae* during nitrogen regulation [65]. The highest levels of *Pr1* were observed in blastospores exposed to live *Aedes* larvae or during host colonisation suggesting that the fungus was responding to host cues before and during infection, with the level of expression remaining constant. Interestingly, *Pr1* levels were depressed in dead hosts whereas *Pr2* levels were highly elevated suggesting that *Pr2* may have functions other than cuticle degradation. The high level of expression of all the virulence related genes examined in blastospores incubated with live *Ae*. *aegypti* larvae suggests that the blastospores can sense the presence of the insect host even before adhering to the cuticle.

Just as the fungus was able to sense the insect before initiating infection, mosquito larvae could also sense the pathogen since AMP genes were elevated 0 hrs pi with only defensin A and B remaining elevated at 12 hrs pi when many insects were highly infected and even dying. In contrast, expression of genes involved with stress management showed an unusual pattern; expression was high at 0 and 22.5hrs pi but declined at 12 hrs pi. The only exception was *Hsp70* which peaked at 12 hr pi. Investment in stress management was a high priority and remained high even at the point of death. Presumably, this was a better investment of resources than the AMPs since most of these are known to exhibit antibacterial rather than antifungal activity [63].

This study shows that the physiological and morphological adaptations of blastospores enable them to adhere to and infect mosquito larvae *via* the integument as well as invade through the gut following ingestion. These multiple routes of entry result in *Ae*. *aegypti* larvae being killed within hours. Blastospores unlike conidia produce copious mucilage which ensures strong adhesion to the host surface. Furthermore, blastospores are not dependent upon differentiation of appressoria or production of Pr1 for infection. These adaptations may explain why blastospores are more virulent than conidia at least when infecting mosquito larvae. However, there are other attributes which make this form of inoculum ideal for control of *Ae*. *aegypti* larvae. They are inexpensive and rapidly produced in liquid media. Their hydrophilic nature means that surfactants are not required. Aerial conidia not only take longer to produce but need surfactants to suspend them in water. Thus, *M*. *brunneum* blastospores meet most of the commercial criteria for selecting pest control products i.e. being relatively inexpensive, safe to humans and the environment, easy to use and highly efficacious (fast acting) against the target pest. These criteria are particularly important since mosquito control often requires treatment of large areas and most affected countries have limited resources.

## Materials and Methods

### Mosquito source and maintenance

*Aedes aegypti* (strain AeAe) eggs, obtained from the London School of Hygiene and Tropical Medicine (UK), were hatched in tap water. Larvae were fed rabbit food (Burgess) and fish food (Tetra pro) and kept at room temperature (25±2°C) in a 16L:8D photoperiod.

### Fungal strains and production

*Metarhizium brunneum* (Formerly *M*. *anisopliae*) isolate ARSEF 4556, identified as highly pathogenic to mosquitoes [24, 47], was maintained on Sabouraud dextrose agar (SDA). Conidia used in assays had over 95% viability. Conidia were harvested from 15 day old sporulating cultures and suspended in 0.03% (v/v) aqueous Tween 80. Blastospores were produced in Adamek’s medium [66]. The medium was inoculated with 10^7^ conidia ml^-1^ and incubated at 27°C in a rotary shaker at 130 rpm for 3 days. Blastospores were harvested by filtering through 2 layers of lens cleansing tissue (Whatman No.105), washed twice with sterile distilled water then centrifuged at 5000 rpm for 10 min (IEC-Centra-3E) and the pellet was suspended in sterile distilled water. Conidia and blastospore concentrations were determined using an improved Neubauer haemocytometer.

### Virulence of *M*. *brunneum* blastospores and conidia

Assays were conducted to compare the virulence of *M*. *brunneum* ARSEF 4556 blastospores and conidia against *Ae*. *aegypti* (L_3-4_) larvae. Cohorts of ten larvae (n = 30) per incubated in 280 ml plastic beakers containing 100 ml of distilled water + fungus used at a final concentration of 10^7^ propagules ml^-1^. Conidia were applied either as a dry powder dusted over the water surface (dry conidia) or as a suspension in 0.03% aqueous Tween 80 (wet conidia). Blastospores were applied as a suspension in distilled water. Larval mortality was recorded every 24 hours for 7 days. Dead larvae were transferred to Petri dishes lined with moist filter paper and incubated at 27°C to encourage fungal emergence and sporulation. Controls consisted of either distilled water or 0.03% aqueous Tween 80. Each treatment was replicated 3 times and the complete experiment was repeated three times. Assays were performed at 25 ± 2°C.

### Role of proteases in pathogenesis

Since conidial proteases play a key role in mosquito larval pathogenesis [47], several complementary studies were conducted to determine if blastospore proteases also played a role in pathogenesis. The first study consisted of virulence bioassays as outlined above being conducted at 20°C and 27°C, since proteolytic activity increases with temperature. The assay was repeated 3 times.

Another study was conducted using protease inhibitors in 24 well plates (Nunc, Roskide, Denmark, n = 72) with one larva per well. Treatments included larvae exposed to 1 ml of 10^7^ blastospores under the following conditions: (1) live blastospores, (2) heat killed blastospores (autoclaved for 15 min at 121°C), (3) live blastospores incubated with chicken egg white Pr1 protease inhibitor (0.1 mg/ml), (4) live blastospores incubated with α2-macroglobulin (1 μg/ml). The latter is an inhibitor of serine, cysteine and metallo-proteases. The inhibitors were purchased from Sigma-Aldrich. Control larvae were exposed to 1ml of distilled water or the inhibitors in distilled water. Mortality was recorded daily over 4 days and the assays repeated 3 times. For more details of the assays see Text S1 in S1 File.

### Infection processes of *M*. *brunneum* blastospores

Larvae were inoculated with blastospores as described above and examined at 12 and 24 hrs post inoculation (pi). Infected larvae were examined by light microscopy to determine if there were preferential sites for blastospores adhesion and to observe the blastospores in the gut. Larvae were also prepared for examination by transmission electron microscopy (TEM). Full details on the preparation and staining of material for TEM are provided in Text S2 in S1 File. Thick sections of the resin embedded larvae were also examined using a light microscope. Full details on the preparation and staining of the material for light microscopy are provided in Text S3 in S1 File. Larvae (n = 20) were also examined by cryo-scanning electron microscopy (SEM) using a Hitachi S4800 field emission microscope equipped with a Quorum PPT2000 cryogenic stage and preparation chamber. Full details on the cryo-SEM are provided in Text S4 in S1 File.

### Blastospore structure

In addition to SEM and TEM, certain cell attributes of blastospores were investigated using a range of fluorochromes. Calcofluor White, Filipin, Rhodamine 123 and DAPI (4’, 6-diamidino-2-phenylindole) were used to visualize β-glucans (in cell walls, septa, mucilage), ergosterol (in cell membranes), mitochondria and nucleic acids, respectively. The fluorochromes were purchased from Sigma. Stained cells were examined in a Zeiss AxioCam MR3 fluorescence microscope. Details on the preparation, staining and visualization of the cell components are provided in Text S5 in S1 File.

### Transcript quantification of insect and fungus-derived genes

#### Samples, RNA extraction and cDNA synthesis

Studies focussed on specific genes which are known to play a major role in fungal pathogenesis or insect defence against infection [63]. Full details of the transcript quantification are given in Text S6 in S1 File. Briefly, *Ae*. *aegypti* larvae (L_3–4_) (n = 10 larvae per replicate with 3 replicates per treatment) were exposed to blastospores of *M*. *brunneum* ARSEF 4556, controls included larvae not exposed to fungus (negative control), as well as a terrestrial insect, *Tenebrio molitor*, that was exposed to blastospores (positive control). Biological samples were frozen in liquid nitrogen and stored at −80°C until required. All samples were ground with a micropestle and total RNA extractions carried out using the RNeasy Micro kit (Qiagen) following the manufacturer's instructions. RNA concentration and purity was assessed at 260 and 280 nm absorbance using a Nanophotometer (Implen). Total RNA (1 μg) was treated with RNase-Free DNase Set (Qiagen) and reverse transcribed using a QuantiTect Reverse Transcription kit (Qiagen) with gDNA elimination reaction, for the experiment to quantify insect-derived transcripts and fungus-derived transcripts respectively. Relative quantification of cDNA was performed by PCR using two reference genes for insect or fungal cDNA samples to ensure consistency between values. For *Ae*. *aegypti*: ribosomal S7 (accession number: AAEL009496) and ribosomal protein 49/L32 (AAEL003396). For *M*. *brunneum*: 18S rRNA and elongation factor tEF (Table S1 in S1 File).

#### Quantitative real time PCR (qRT-PCR)

PCR reactions were performed in 10 μl volumes consisting of 1μM of each primer, 2 μl of the cDNA sample, 5 μl SYBR Green Fastmix (Quanta) and 1 μl ultrapure water. All reactions were carried out in triplicate. PCR cycling conditions were as follows: one cycle of 45°C for 5 min and 95°C for 3 min followed by 39 cycles of 95°C for 10 sec, 60°C for 10 sec and 72°C for 30 sec. A dissociation step of 65–95°C over 5 sec was used for melt curve analysis to detect non-specific products in the reaction. The reaction was performed using a Rotor-Gene 6000 (Corbett Research). The comparative Ct method (2-∆∆Ct) was used to calculate relative gene expression [67].

### Statistics

Mosquito larval survival was investigated in three treatment groups; 1) conidia, 2) blastospores and 3) blastospores incubated in protease inhibitors. Each experimental treatment was replicated three times. The whole study was repeated three times. Cumulative survival was quantified using Kaplan-Meier plots. Pairwise comparison were made between; 1) conidia vs blastospores and 2) blastospores vs blastospores with inhibitor using log-rank tests. *Pr1* activity and molecular data sets were analysed using one-way Analysis of Variance (ANOVA) with Tukey´s HSD *post-hoc* test to assess pairwise comparisons.

Prior to analysis, gene expression data was logarithm transformed, conforming to ANOVA assumption of homogeneity of variance [68]. All statistical analyses were carried out using SPSS v22.0 [69], Rstudio Version 0.99.482 [70]and GraphPad Prism v5.0 (GraphPad Software, USA).

### Genes and corresponding accession numbers (see Text S6 in S1 File)

*Ae*. *aegypti* genes; Ribosomal S7 (AAEL009496), Ribosomal protein 49/L32 (AAEL003396), Defensin A (AAEL003841), Defensin B (AF156090.2), Defensin D (AAEL003857), Cecropin A (AAEL000627), Cecropin G (AAEL015515), heat shock protein 70 (AAEL016995), heat shock protein 83 (AAEL011704), Thiol peroxidase (AAEL004112), Cytochrome P450 (AAEL009123), and Glutathione peroxidase (AAEL008397)

*M*. *brunneum;* PR1a (MBR_01491), PR2 (MBR_06579), MAD1 (MBR_08250), MAD2 (DQ338438.1), MOS1 (MBR_07375), Cag 8 (MBR_00569), Nrr1 (MBR_08301), 18s (DQ288247.1),Translation elongation factor (MBR_08275)

## Supporting Information

S1 File **Text S1: Enzyme and enzyme inhibitor assays.** Text S2: Transmission electron microscopy. Text S3: Light microscopy of resin embedded sections. Text S4: Cryo-SEM. Text S5: Fluorescence Microscopy. Text S6: Transcript quantification of insect and fungus-derived genes.
(DOC)
