# Supplementary material for: Metarhizium brunneum Blastospore Pathogenesis in Aedes aegypti Larvae: Attack on Several Fronts Accelerates Mortality
Source: PLoS Pathog. 2016 Jul 7;12(7):e1005715. doi: 10.1371/journal.ppat.1005715 (PMC4936676; doi:10.1371/journal.ppat.1005715)
Supplement: S1 File — Text S1: Enzyme and enzyme inhibitor assays. Text S2: Transmission electron microscopy. Text S3: Light microscopy of resin embedded sections. Text S4: Cryo-SEM. Text S5: Fluorescence Microscopy. Text S6: Transcript quantification of insect and fungus-derived genes. (DOC) [file ppat.1005715.s001.doc]

**SUPPORTING INFORMATION TEXT**

##### Experimental Procedures

**S1 Enzyme and enzyme inhibitor assays**

**(i) Pr1 protease activity.** Pr1 activity bound to conidia was assessed using methods adapted from Shah *et al*. . Briefly, 10 mg of spores were incubated in 1 ml of 0.1 M Tris–HCl (pH 7.95) containing 1 mM Succinyl-Ala-|Ala-Pro–Phe-p-nitroanilide (Sigma) for 5 min at room temperature. After incubation, the suspension was centrifuged at 12,000 x g (Sanyo, Harrier 18/80 centrifuge) for 5 min. The supernatant (200 µl) was transferred to a 96-well plate (Greiner Dio-One) and absorbance measured using a BioTek Synergy H1 multi-mode reader at 405 nm. Buffered substrate was used as a control.

**(ii) Protease inhibition assays.** *Ae. aegypti* larvae were exposed to conidia (1x10^7^ conidia ml^-1^) of *M. brunneum* ARSEF 4556 suspended in 1 ml of 0.03% aqueous Tween 80 containing either chicken egg white (specific Pr1 type enzyme inhibitor) inhibitor (0.1 mg/ml) or α2-macroglobulin inhibitor (1 µg/ml) a global protease inhibitor (serine, cysteine, metallo-). All the inhibitors were purchased from Sigma-Aldrich. Controls consisted of distilled water or inhibitor in distilled water. Larval mortality was recorded daily over a 4 day period. All assays were repeated three times.

**S2 Transmission electron microscopy**

Larvae at various stages of infection and controls (uninfected) were fixed overnight in a solution consisting of 3% (v/v) glutaraldehyde, 1% acrolein and 0.1% CaCl_2_ in 0.1M PIPES pH 7.4, at 4˚C. The larvae were washed 4 times with 0.2M PIPES pH 7.4 + 0.1% CaCl_2_ at 20 minute intervals then post-fixed in 1% (w/v) osmium tetroxide in 0.1% CaCl_2_ in 0.15 M PIPES buffer pH 7.4 for 1 hr at room temperature. The specimens were washed 3 times with filtered dH_2_O at 20 min intervals before being immersed for 1 hr in 2% (w/v) aqueous uranyl acetate in the dark. The specimens were dehydrated though a graded acetone series (50, 70, 80, 90, 95%) every 20 min before being transferred to 100% dry acetone for 30 mins at RT with five changes during this period. The specimens were then suspended in a graded series of acetone and Spurr’s resin (3:1 for 1 hr, 1:1 overnight, 1:3 for 1 hr, 100% resin for 6 hrs on a stirrer, 100% resin overnight). Samples were embedded in 100% Spurr's resin and polymerized at 70 °C for 8 hrs. Ultrathin (gold) sections were cut using a Reichert ultramicrotome, mounted onto copper grids and examined in a JEOL- JEM-1200XII transmission electron microscope (TEM) at 120 kV.

**S3 Light microscopy of resin embedded sections**

Specimens were prepared as described above and thick sections cut with a Reichert ultramicrotome, stained with general Toluidine Blue cell stain (Microscope Services UK) and examined using a light microscope (Nikon Eclipse 90i). Images were recorded using a digital camera.

**S4 Cryo-SEM**

The larvae were mounted on SEM sample holders using cryogenic glue, both horizontally for imaging of the cuticle and vertically for fracturing and cross sectional imaging before being plunged into a nitrogen slush for rapid freezing and then transferred to the cryogenic preparation chamber under vacuum. The specimens were warmed up to -90°C for 10 minutes to remove surface ice then returned to -130°C. Fracturing of the vertically mounted larvae was performed inside the preparation chamber at -130°C using a rotating knife. The specimens were then coated with approximately 5nm of Platinum then transferred to the SEM stage. Imaging took place at -130°C.

**S5. Fluorescence Microscopy (all reagents were purchased from Sigma unless indicated otherwise)**

***Cell wall staining.*** The vital fluorochrome, Calcofluor white, binds to β-glucans found in fungal cell walls, septa and mucilage. An aqueous stock solution of 1 mg/ml Calcofluor white was stored in the dark at 4°C until required. Blastospores were harvested and pelleted in a centrifuge, washed twice with distilled water with intervening centrifugation steps, resuspended in 0.01% Calcofluor and incubated overnight at room temperature in the dark. The spores were pelleted and washed three times with distilled water as described earlier to remove unbound dye. Stained blastospores were examined in a Zeiss fluorescence microscope using excitation and emission filters of 350nm and 435nm, respectively.

***Plasma membrane sterol staining.*** Filipin is a sterol specific dye which facilitates investigation of fungal membrane dynamics . A stock solution of filipin (5 mg ml^-1^ in DMSO) was prepared and stored at -20 ̊C in the dark and used within 2 weeks. The working solution was prepared by diluting the stock solution 1:100 in 10mM phosphate-buffered saline (PBS) pH 7.2 and added to the blastospore pellet (concentration 10^7^ conidia ml^-1^). After 1 hr incubation at RT, the blastospores were washed twice with PBS then observed immediately in a Zeiss fluorescence microscope using the appropriate excitation (340-380nm) and barrier (385-470nm) filters.

***DNA staining.*** DAPI (4’, 6-diamidino-2-phenylindole), a DNA specific fluorochrome, was used to determine the number and distribution of nuclei in blastospores. A stock solution was prepared by dissolving 5mg of DAPI in 1 ml of deionized water (14.4 mM), heated in a water bath at 47 ˚C until it dissolved. The stock solution could be stored at 2-6 ˚C for up to 6 months or at ≤ -20 ˚C for longer period. Intermediate dilution was prepared by adding 2.1 µl of stock solution to 100 µl PBS (300 µM). To prepare the working solution, the intermediate solution was diluted 1:1000 in PBS to give a 300 nM working solution. Blastospores were washed twice with sterile dH_2_O and once with PBS before suspending the pellet in 1ml of the working solution at a concentration of 10^7^ conidia ml^-1^. After incubation for 1-5 min at RT in the dark, the pellet was washed twice with PBS to remove unbound dye and examined in a Zeiss fluorescence microscope using excitation and barrier filters of 306-370nm and 420-460nm respectively.

***Mitochondria.*** These were observed using the potentiometric dye Rhodamine 123. A 25mM stock solution was prepared by dissolving 1 mg of Rhodamine in 105 µL of DMSO (25 mM). A working solution (500µM) was prepared by diluting the stock solution 1:50 in DMSO i.e. 10 µL stock solution in 490 µL DMSO. Blastospores were washed twice with sterile dH_2_O and re-suspended in 0.1M sodium citrate buffer, pH 5.8. Ten µL of Rhodamine 123 was added to 90µL of the suspension, incubated for 1 hr in the dark, and then centrifuged for 1 min at 10000g. The supernatant was discarded and the blastospores were washed with PEM buffer, which consists of 0.1 M 1,4-Piperazinediethanesulfonic acid (PIPES) pH 6.96 plus 2mM Ethylene-bis(oxyethylenenitrilo)tetraacetic acid (EGTA) and 1mM MgSO_4_. Cells were examined in a Zeiss fluorescence microscope using excitation and barrier filters of 511 nm and 534 nm, respectively.

**S6 Transcript quantification of insect and fungus-derived genes**

**(i) *Samples, RNA extraction and cDNA synthesis:*** *Ae. aegypti* larvae (L_3-4_) were exposed to 10^7^ blastospores ml^-1^ of *M. brunneum* ARSEF 4556. Fungus free controls were also included. For the transcript analysis of insect-derived genes, ten larvae were placed in 10 ml distilled water in 6-well Nunc cell culture multidishes (128 x 86 cm, Thermo Scientific) and incubated for 20.5 hrs at room temperature. Six replicates were harvested immediately before treatment (time 0) and after 12 and 20.5 hrs , dead larvae were removed and the remaining live insects were pooled and divided into batches of 10 per sample and frozen in liquid nitrogen and stored at -80°C until required.

For the transcript analysis of fungus-derived genes, *Ae. aegypti* larvae were exposed to ARSEF 4556 blastospores as described above for 20.5 hr at room temperature. The larvae were divided into living and dead samples, frozen in liquid nitrogen and stored at -80°C until required. Other samples included: *M. brunneum* ARSEF 4556 blastospores that been exposed to *Ae. aegypti* larvae for 20.5 hrs , blastospores incubated for same time period but in the absence of larvae, and a terrestrial host (*Tenebrio molitor*) positive control. The latter was exposed to the same concentration of blastospores but incubated on moist filter paper for 20.5 hr at room temperature. Blastospores were collected by centrifugation at 18,000 xg for 5 min in a centrifuge (MICRO 22). Each sample was frozen in liquid nitrogen, freeze dried and stored at -80°C until required. Three replicate treatments were carried out for each sample.

Samples were ground with a micropestle and total RNA extractions carried out using the RNeasy Micro kit (Qiagen) following manufacturer’s instructions. A RNA carrier was also used for samples from the experiment to measure *Ae. aegypti*-derived genes according to manufacturer’s instructions (Qiagen). RNA concentration and purity was assessed at 260 and 280 nm absorbance using a Nanophotometer (Implen).

**(ii) Quantitative PCR (qPCR):** Optimal primer concentration and annealing temperature were determined using samples expected to contain the genes of interest. qPCRs were performed in 10 μl reactions (2 μl diluted cDNA, 1 μl optimised concentration of each primers and 5 μl PerfeCta® SYSYBR® Green Fast mix (Quanta) for the Rotor-Gene 6000 system).

For *Ae. aegypti* genes *ada-rb*7, *ada-rp*49, *ae-def*A, *ae-def*B, *ada-ccg*, *ae-ca2,* *hsp*70, *hsp*83, *cyp*6z6, *tpx* and *gpx*, the cycling conditions were: 95°C 5 minutes, 42 cycles 95°C 5 sec, primer annealing (65-59°C) 10 sec, 72°C for 20 sec. A touchdown profile was used for *ada-def*D and *aec*A2 where the annealing temperature was reduced from 65°C by 1°C per cycle for the first 5 cycles. For *M. brunneum* genes *18S rRNA*, *tef*, *Pr1*A, *Pr2*, *nrr1, cag8, MOS1*, *Mad1* and *Mad2* cycling conditions were: 45°C 5 min, 95°C 3 min, and 39 cycles of 95°C 10 sec, 60°C 10 sec and 72°C 30 sec. A high resolution melt curve (HRM) analysis was included for all reactions to ensure no spurious products were present. Non-template water was used as a negative control in each run to ensure no contamination was present in the PCR reagents. A two-fold dilution series of pooled cDNA samples (at a concentration of 32, 16, 8, 4, 2, and 1 ng/ml) was used for each run to provide a standard curve for each primer. The efficiency of RT-PCR was calculated by generating a standard curve from plotting ct values against the log template amount, and then using the slope of the standard curve. The following formula was used to calculated the efficiency: PCR efficiency (%) = 10^(-1/5)^ -1) × 100. Samples with Ct (cycle threshold) values higher than non-template controls were considered as zero. The samples were run in triplicate and replicates were run in duplicate.

|  | Table S1. *Metarhizium brunneum* and *Aedes aegypti* loci used for expression analysis | | | | |  |  |
| --- | --- | --- | --- | --- | --- | --- | --- |
| **Putative function** | | **Locus** | **Accession number** | **Primer name** | **Forward primer** | **Reverse Primer** | **Sequence reference** |
| Housekeeping (*Ae. aegypti*) | | Ribosomal S7 | AAEL009496 | Ada-Rb7 | TCAGTGTACAAGAAGCTGACCGGA | TTCCGCGCGCGCTCACTTATTAGATT |  |
|  |  | Ribosomal protein 49 | AAEL003396 | Ad_RP49 | ACAAGCTTGCCCCCAACT | CCGTAACCGATGTTTGGC |  |
|  |  | /L32 |  |  |  |  |  |
| Antimicrobial peptides (*Ae. aegypti*) | | AeDA, Defensin A | AAEL003841 | AeDA | CCGAAAGGACCAACCATGAA | ATTCCGACAGACGCACACCCT |  |
|  |  | AeDB, Defensin B | AF156090.2 | AeDB | TCATTTGTTTCCTGGCTCTGTG | GCGGCCTGATAGGTTTCCTC |  |
|  |  | Ada-Defensin D | AAEL003857 | Ada-DefD | CGGTGCTGGCGGACGAA | GCAATGAGCAGCACAAGCACTATC |  |
|  |  | Cecropin A | AAEL000627 | AeCA2 | TGGCTGTTCTTCTCCTGA | AAAACTCGTTTTCCTGCAC |  |
|  |  | Cecropin G | AAEL015515 | Ada-CcG | TCACAAAGTTATTTCTCCTGATCG | GCTTTAGCCCCAGCTACAAC |  |
| Stress | | Hsp70, heat shock protein 70 | AAEL016995 | Hsp70 | CCCGTCCTACGTGGCGTTCA | GGTGGCCTGACGTTGCGAGT |  |
| (*Ae. aegypti*) | | Hsp83, heat shock protein 83 | AAEL011704 | Hsp83 | AAGGCCGTTAAGGATCTGGT | CGCTAGTGTGGGGAAGAGAG |  |
| Reactive oxygen | | TPX, Thiol peroxidase | AAEL004112 | TPX2 | TCGACCGACAGTCACTTCAC | CTGGCGGAGATTCTGCTTAC |  |
| (*Ae. aegypti*) | | CYP6Z6, Cytochrome P450 | AAEL009123 | CYP6Z6 | CTGCCTTATTTGGACTTATGC | ATCACAACACTGGATTCTGG |  |
|  | | GPX, Glutathione peroxidase | AAEL008397 | GPX | ATATGGCGAAACGGAAGGTC | TCCCCGTTGACGTATATCTTG |  |
| Proteases (*M. brunneum*) | | PR1a | MBR_01491 | PR1a | GATTGGTGGCAGCACTAAC | TCCTGGATCTTCTTGCAAAG |  |
|  |  | PR2 | MBR_06579 | PR2 | TACGCCACATTGCCAGA | GCATGTCGCACGATCAA |  |
| Adhesion genes (*M. brunneum*) | | MAD1 | MBR_08250 | MAD1 | CTCCTCACATCACCCAGGTT | GGGAGTAGGCATGACGATGT |  |
|  |  | MAD2 | DQ338438.1 | MAD2 | CTATGTCCACCCTTGCGACT | AGCACAGCTGATGAGGGTCT |  |
| Osmotic stress (*M. brunneum*) | | MOS1 | MBR_07375 | MOS1 | ACTGCCTGACAAGGACAACC | CATTGCTTGGTTATGCATCG |  |
| Regulator of G-protein signaling (*M. brunneum*) | | Cag 8, Conidiation-associated gene | MBR_00569 | Cag8 | AAGCTGATGGCTAGCGATTC | TTGCGGTTGGAACGACTTTG |  |
| Regulator of nitrogen (*M. brunneum*) | | Nrr1 | MBR_08301 | Nrr1 | ACTATTGATGAGCGTCGTAAC | TGCGTCGTTGTCCATGAAG |  |
| Housekeeping genes (*M. brunneum*) | | 18s | DQ288247.1 |  | CGAAAGTCGCAATGGCTCA | CCGAAGTCGGGATTTTTAGC |  |
|  |  | Translation elongation factor | MBR_08275 | MaHKtEF | CGAGCGTGAGCGTGGTA | CAGCCTCGAACTCACCAG |  |

**SUPPORTING INFORMATION REFERENCES**

1. Shah FA, Wang CS, Butt TM. Nutrition influences growth and virulence of the insect-pathogenic fungus *Metarhizium anisopliae*. FEMS Microbiology Letters. 2005;251(2):259-66.

2. Van Leeuwen M, Smant W, De Boer W, Dijksterhuis J. Filipin is a reliable in situ marker of ergosterol in the plasma membrane of germinating conidia (spores) of *Penicillium* *discolor* and stains intensively at the site of germ tube formation. Journal of Microbiological Methods. 2008;74(2):64-73.

3. Telang A, Qayum A, Parker A, Sacchetta B, Byrnes G. Larval nutritional stress affects vector immune traits in adult yellow fever mosquito *Aedes aegypti* (Stegomyia aegypti). Medical and Veterinary Entomology. 2012;26(3):271-81.

4. Bonizzoni M, Dunn WA, Campbell CL, Olson KE, Dimon MT, Marinotti O, et al. RNA-seq analyses of blood-induced changes in gene expression in the mosquito vector species, *Aedes aegypti.* BMC Genomics. 2011;12(1):82.

5. Butt TM, Greenfield BP, Greig C, Maffeis TG, Taylor JW, Piasecka J, et al. *Metarhizium anisopliae* Pathogenesis of Mosquito Larvae: A Verdict of Accidental Death. PloS One. 2013;8(12):e81686.

6. Xi Z, Ramirez JL, Dimopoulos G. The *Aedes aegypti* toll pathway controls dengue virus infection. PLoS Pathog. 2008;4(7):e1000098.

7. Muturi EJ, Kim CH, Alto BW, Berenbaum MR, Schuler MA. Larval environmental stress alters *Aedes aegypti* competence for Sindbis virus. Tropical Medicine & International Health. 2011;16(8):955-64.

8. Zhao L, Becnel JJ, Clark GG, Linthicum KJ. Expression of AeaHsp26 and AeaHsp83 in *Aedes aegypti* (Diptera: Culicidae) larvae and pupae in response to heat shock stress. Journal of Medical Entomology. 2010;47(3):367-75.

9. Pan X, Zhou G, Wu J, Bian G, Lu P, Raikhel AS, et al. *Wolbachia* induces reactive oxygen species (ROS)-dependent activation of the Toll pathway to control dengue virus in the mosquito *Aedes aegypti.* Proceedings of the National Academy of Sciences. 2012;109(1):E23-E31.

10. Fang W, Bidochka MJ. Expression of genes involved in germination, conidiogenesis and pathogenesis in *Metarhizium anisopliae* using quantitative real-time RT-PCR. Mycological Research. 2006;110(10):1165-71.

11. Wang C, Duan Z, Leger RJS. MOS1 osmosensor of *Metarhizium anisopliae* is required for adaptation to insect host hemolymph. Eukaryotic Cell. 2008;7(2):302-9.

12. Carneiro-Leão M, Andreote F, Araújo W, Oliveira N. Differential expression of genes involved in entomopathogenicity of the fungi *Metarhizium anisopliae* var. anisopliae and *M. anisopliae* var. acridum (Clavicipitaceae). Genetics and Molecular Research. 2011;10(2):769-78.
